# INTERSECTION OF RACE AND PLACE-BASED DISPARITIES AMONG 65+ IN MISSISSIPPI

**DOI:** 10.1093/geroni/igad104.2347

**Published:** 2023-12-21

**Authors:** Taylor Jansen, Yan-Jhu Su, Chae Man Lee, Nina Silverstein, Elizabeth Dugan, Kina White

**Affiliations:** University of Massachusetts Boston, Boston, Massachusetts, United States; University of Massachusetts Boston, Boston, Massachusetts, United States; University of Massachusetts Boston, Boston, Massachusetts, United States; University of Massachusetts Boston, Boston, Massachusetts, United States; University of Massachusetts, Boston, Boston, Massachusetts, United States; Mississippi State Department of Health, Ridgeland, Mississippi, United States

## Abstract

Disparities in healthy aging have been attributed to race, gender, SES, and zipcode. The Mississippi Healthy Aging Data Report (HADR) is a new resource in the state to advance age-friendly progress reporting 150 indicators related to healthy aging for 82 counties. The present study explored how race and place may be associated with community health outcomes. The main data sources include the American Community Survey, the Behavioral Risk Factor Surveillance System, and the Centers for Medicare and Medicaid Services. Bivariate statistical analyses and mapping were conducted to compare the 65+ population in Mississippi living in rural (N=65) and urban areas (N=17); counties with high (N=44) and low (N=38) proportions of 65+ black residents; and high and low black populations by rural (high: N= 38; low: N=27) and urban counties (high: N=6; low: N=11). Bivariate analyses found that the rural counties with high proportions of 65+ black residents reported consistently worse rates for 26 out of 28 indicators of healthy aging. ArcGIS Mapping demonstrated that the Mississippi rural, black population tended to be centralized to the Delta region, a historically impoverished area. This population reported the highest rates of chronic conditions and poverty, lowest rates of education and life expectancy, and the lowest access to primary care providers and hospitals. This study underscores the importance of data tools like HADR to improve healthy aging for everyone. Understanding the distribution of community rates makes disparities evident and may help practitioners and policymakers to allocate resources to areas of highest need.

